# Effect of Fabrication Parameters on the Performance of 0.5 wt.% Graphene Nanoplates-Reinforced Aluminum Composites

**DOI:** 10.3390/ma13163483

**Published:** 2020-08-07

**Authors:** Shu Mei Lou, Chuan Dong Qu, Guang Xin Guo, Ling Wei Ran, Yong Qiang Liu, Ping Ping Zhang, Chun Jian Su, Qing Biao Wang

**Affiliations:** 1 Department of Mechanical and Electrical Engineering, Shandong University of Science and Technology, Qingdao 266590, China; quchuandong@163.com (C.D.Q.); 17860783376@163.com (G.X.G.); r18306440915@163.com (L.W.R.); Mr_liuyongqiang@163.com (Y.Q.L.); ZPP542968@163.com (P.P.Z.); suchunjian@sdust.edu.cn (C.J.S.); 2 National Engineering Laboratory for Coalmine Backfilling Mining, Shandong University of Science and Technology, Tai’an 271019, China

**Keywords:** GNP, graphene nanoplates-reinforced aluminum composite, orthography test, parameter optimization

## Abstract

Aluminum composites reinforced by graphene nanoplates(GNP) with a mass fraction of 0.5% (0.5 wt.% GNP/Al) were fabricated using cold pressing and hot pressing. An orthogonal test was used to optimize the fabrication parameters. Ball milling time, ball milling speed, and ultrasonic time have the largest influence on the uniformity of the graphene in the composites. Afterwards, the microstructure, interfacial properties, and fracture morphology of the composites obtained with different parameters were further analyzed. The results show that ball milling time and ball milling speed have obvious influences on the mechanical properties of the composite. In this paper, when the ball milling speed is 300 r/min and the ball milling time is 6 h, the dispersion uniformity of graphene in the 0.5 wt.% GNP/Al composite is the best, the agglomeration is the lowest, and the mechanical properties of the composites are the best, among which the tensile strength is 156.8 MPa, 56.6% higher than that of pure aluminum fabricated by the same process (100.1 MPa), and the elongation is 19.9%, 39.8% lower than that of pure aluminum (33.1%).

## 1. Introduction

Aluminum is a structural material used commonly in aerospace, automobile, machinery, electronics, and other fields because of its light weight, high strength, good ductility, easy processing, and so on [1,2,3]. The development of science and technology has higher requirements for the quality and performance of materials, so traditional Al and alloys no longer meet the needs of modern society [4]. Graphene is an ideal reinforcement for metal matrix composites due to its good friction properties [5,6,7,8,9], high conductivity [10,11], high thermal conductivity [12,13], good mechanical properties [14,15,16,17,18,19,20,21,22], and so on. GNP (graphene nanoplates), composed of a small number of graphene layers, own properties similar to those of monolayer graphite with high yield and low price. In addition, GNP retains some residual oxygen groups, which have a positive effect on the mechanical properties as well as the interfacial structure of the composites.

In recent years, research on graphene-reinforced Al composites has gradually increased. Yan et al. [16] used ball milling and the hot isostatic pressing method to prepare 0.5 wt.% GNP/Al composites. GNP and Al were put into a ball milling tank, the ball milling speed was 75 r/min, the ball-to-powder weight ratio (B-P ratio) was 10:1, the ball milling time was 12 h, and the dried composite powder was hot isostatically pressed at 480 °C. The results show that graphene is uniformly distributed in the Al alloy matrix and forms a good interface bond with the matrix, and there is no interfacial reaction. Shin et al. [18] prepared few-layer graphene-reinforced Al2024 composites using ball milling and hot rolling. The results show that graphene can significantly improve the strength of the composites. In the process of plastic deformation, graphene can hinder the movement of dislocations and contribute to the strengthening of the composites. Bastwors et al. [23] prepared GNP/Al composites with 1.0 wt.% GNP using ball milling and hot pressing, and the effect of the ball milling process on the dispersion of graphene was studied. The results show that ball milling does little damage to the structure of graphene, and the ball milling process is helpful for the uniform dispersal of graphene, reducing the number of graphene layers. R. Perez Bustamante et al. [15] prepared graphene-reinforced composites using mechanical alloying. The milling times were 1 h, 3 h, and 5 h, and the GNP contents were 0.25%, 0.5%, and 1.0%. The results show that GNP was evenly dispersed in the Al matrix, and GNP existed at the grain boundary whose structure remained unchanged.

At present, there are still some problems in the fabrication of graphene-reinforced Al composites, such as how to disperse the graphene uniformly in the Al matrix [24,25], how to reduce the interfacial reaction between graphene and the Al matrix [26], and how to solve the wettability problem between graphene and the Al matrix [27]. Among these problems, achieving a uniform dispersal of graphene in the Al matrix without destroying the integrity of graphene nanoplate is the primary problem that can determine whether the enhancement effect of graphene can be fully utilized. However, most studies focus on the influences of milling time and milling speed [15,23,24,25] on the dispersion of the graphene, and few of them study the influences of the fabrication parameters as a whole systematically.

In this study, graphene nanoplates with a mass fraction of 0.5 were mixed with Al powder using ultrasonic dispersal in ethanol solution and ball milling, and the graphene-reinforced aluminum composites (0.5 wt.% GNP/Al) were prepared via cold pressing and vacuum hot pressing. To maximize the uniform dispersal of graphene in the Al matrix while retaining the integrity of the GNP to the greatest extent, the optimal fabrication parameter combination needs to be determined. Therefore, in this paper, the fabrication parameters were grouped by orthogonal testing, the effects of the five fabrication parameters on the dispersion uniformity of graphene were studied, and the main influential parameters were obtained. The effects of ultrasonication time, ball milling time, and ball milling speed on the mechanical properties or microstructures of the composites were studied, and the optimal parameters that ensure the largest strength of the 0.5 wt.% GNP/Al composites fabricated by the cold pressing and vacuum hot pressing were obtained.

## 2. Experiments

### 2.1. Materials

**Figure 1:**
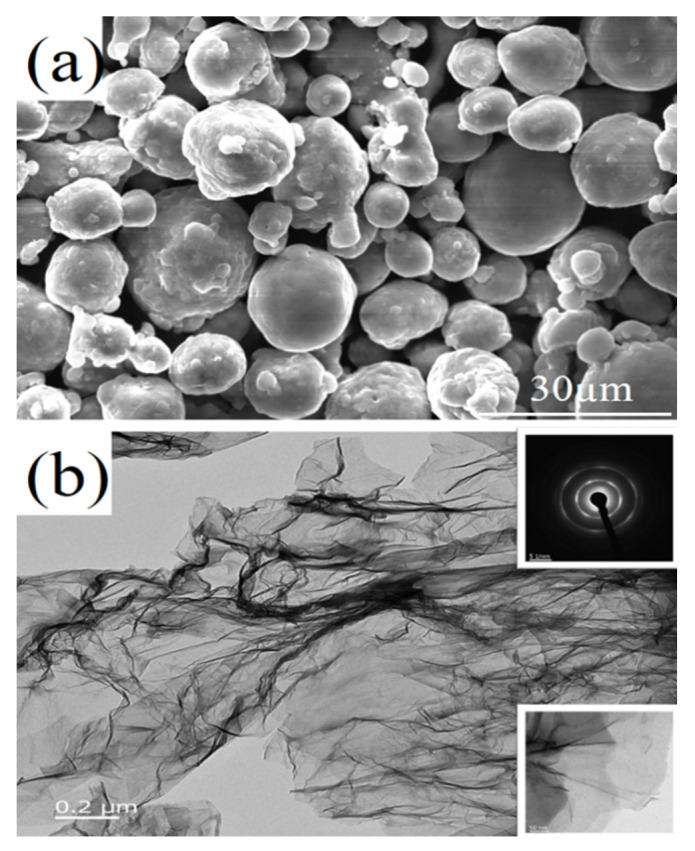
Al powder and GNP. (**a**) SEM of pure Al powder; (**b**) TEM of GNP.

The pure Al powder produced by Shandong Sitaili Metal Materials Co., Ltd. (Jinan, China) was used as the matrix whose particle size is approximately 30 μm; graphene was purchased from Qingdao Huagao Diluted Energy Co., Ltd. (Qingdao, China) with a specific surface area of approximately 400 m^2^/g and approximately 3–5 layers with a particle size of 0.1–5.0 μm. Figure 1 shows the scanning electron microscopy (SEM) image of pure Al powder and the transmission electron microscopy (TEM) image of graphene. The pure Al powder particles are nearly spherical and have a uniform particle size, as shown in Figure 1a. Figure 1b is a TEM image of the graphene. The GNP is feathery and translucent, with typical folded structure characteristics. As seen from Figure 1b in the lower right corner, the number of graphene layers is approximately 3–5. In Figure 1b, the electron diffraction pattern shows that the GNP belongs to a polycrystal, and the formation of the diffraction ring is due to the electron beam hitting the fold of the GNP. The different orientations of the different regions on the fold lead to the mixing of the diffraction spots of different orientations to form the diffraction ring.

### 2.2. Fabrication of the 0.5 wt.% GNP/Al Composite Powder

First, the pre-weighed graphene is mixed with an appropriate amount of industrial ethanol and placed in an ultrasonic cleaner (KQ-300E, Kunshan Ultrasonic Instrument Co., Ltd. Kunshan, China) to reduce the agglomeration of graphene and increase its dispersibility. The ultrasonication time was set to 60 min or 90 min. Then, the alcoholic solution of graphene and Al powder prepared in the ethanol solution dispersion is packaged into a tank of a planetary ball mill (XQM-8, Changsha Tianchuang Powder Technology Co., Ltd., Changsha, China) for ball milling. Stearic acid is used as the process control agent, which can increase the dispersibility and prevent cold welding. Nitrogen is used as a shielding gas to prevent Al powder from being oxidized.

### 2.3. Orthogonal Test Design of the Fabrication Parameters

In this paper, ball milling speed, ball milling time, B-P ratio, ultrasonication time, and stearic acid content were selected as the factors to study. To simplify the experimental process, this paper uses the orthogonal test method to distribute the five fabrication parameters reasonably, and the orthogonal distribution table of parameters (L8 (4 × 24)) is shown in Table 1. To facilitate the expression, the five parameters are named X_1_, X_2_, X_3_, X_4_, and X_5_. As shown in Table 2, X_1_ has 4 levels, while X_2_, X_3_, X_4_, and X_5_ have 2 levels.

**Figure 2:**
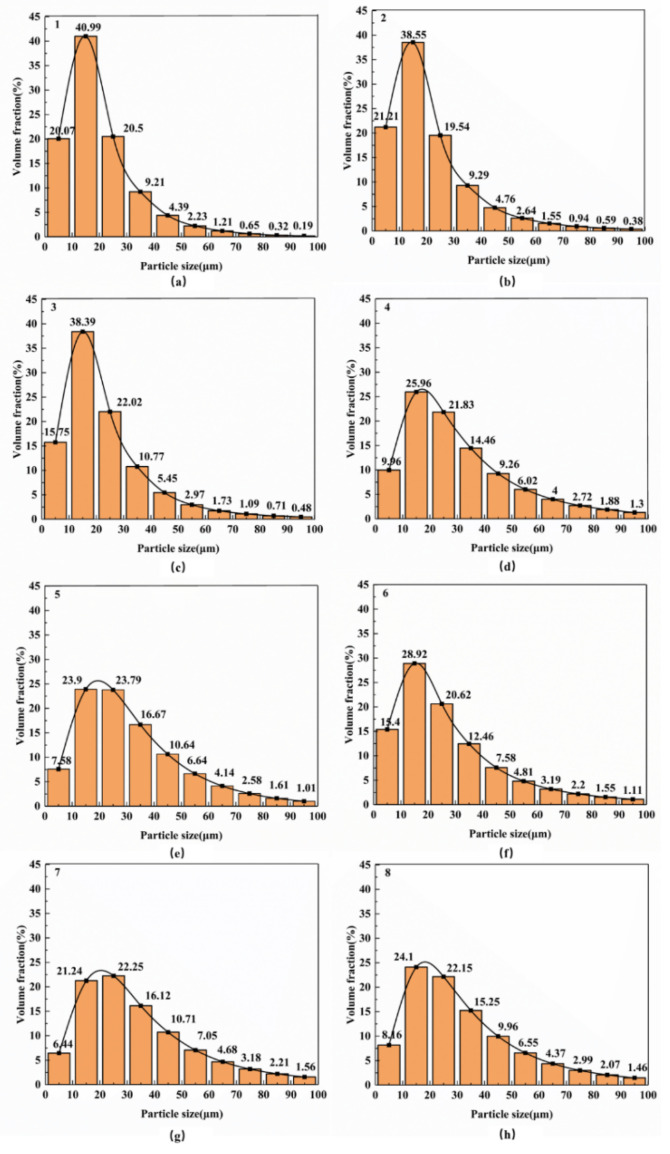
Particle size of 0.5 wt.% GNP/Al powder with different parameters. (**a**,**c**,**e**,**g**): 200 r/min, ball milling time of 2, 4, 6, 8 h; (**b**,**d**,**f**,**h**): 300 r/min, ball milling time of 2, 4, 6, 8 h.

**Figure 3:**
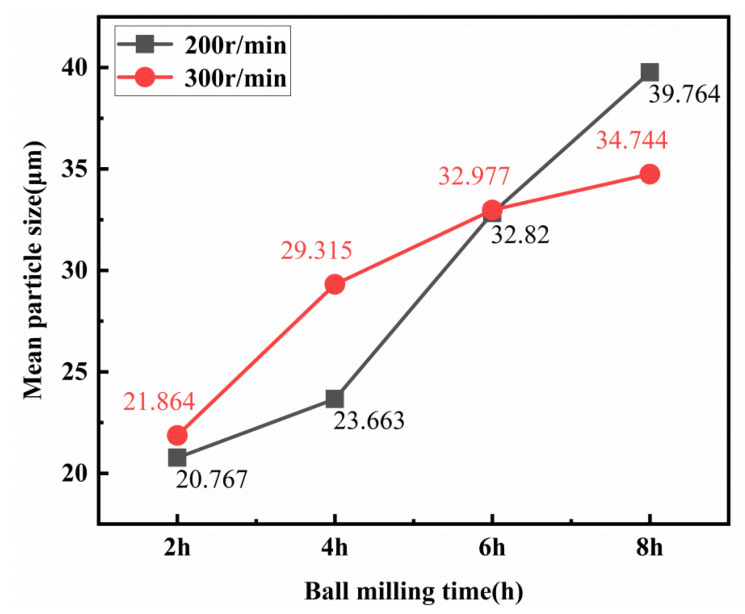
Mean particle size of 0.5 wt.% GNP/Al powder vs. ball milling time.

A particle size analyzer (Hydro-2000 Mu (A), Malvern instruments Ltd., Malvern, UK) was used to test the particle size distribution of the composite powders after ball milling. Figure 2 shows the particle size of the 0.5 wt.% GNP/Al composite powder corresponding to the test numbers 1–8 in Table 1. As the milling time increases, the particle size of the composite becomes increasingly uniform. It can be seen from Figure 3 that, in the process of ball milling, the average particle size of Al powder increases gradually due to cold welding, but this growth tends to slow down gradually. The corresponding degree of slowing for the ball milling speed of 300 r/min is smaller than that for 200 r/min because the impact of the ball on the particles is larger when the ball milling speed is larger, so the cold welding effect is less serious.

The purpose of ball milling is to achieve uniform dispersion of the graphene and uniform particle size of the composite powder. In the initial stage of this paper, the standard deviation value (SDV) of the particle size of the composite powder samples after ball milling, as shown in Figure 2, is used as the optimization objective of the orthogonal test, and the reliability will be analyzed later.

(1) $$\mathrm{SDV}=\sqrt{\frac{\sum_{i=1}^{n}{\left(D_{i}-\bar{D}\right)}^{2}}{n}}$$

where n is the number of particles, $D_{i}$ is the particle size of the composite powder, and $\bar{D}$ is the average value of the composite powder.

The average SDV value of each factor at different levels is calculated to study the influence of the parameters on the dispersal of the composite, as shown in Table 3. It can be seen that there are different trends in the average value of SDV under different factor levels. These trends reflect the influence of different levels of each factor on the particle size homogenization of the composite powder after ball milling. As seen from Table 3, with the increase of ball milling time (X_1_), the average value of SDV first changed from 0.39 (2 h) to a maximum of 2.0 (4 h), decreased to 0.055 (6 h), with a decrease ratio of 97.25%, and then increased to 1.775 (8 h). This shows that the ball milling time has a very obvious effect on the particle size homogenization of the composite powder, and the particle size of the powder is the most uniform when the ball milling time is 6 h. In addition, with the increase in ball milling speed (X_2_), the average value of SDV decreased from 3.52 (200 r/min) to 2.065 (300 r/min), with a decrease of 36.5%, indicating that the ball milling speed had a significant effect on the particle size homogenization of the composite powder, and the speed of 300 r/min could make the particle size more uniform. When the B-P ratio (X_3_) changed, the average value of SDV decreased from 2.90 to 2.675, a decrease of 7.8%, indicating that the ball-to-powder weight ratio has little impact on the particle size homogenization of the composite powder. When the ultrasonication time (X_4_) changed, the average value of SDV increased from 2.19 to 3.4, an increase of 35.6%, which indicates that the ultrasonication time has an obvious effect on the particle size homogenization of the composite powder. When the stearic acid content (X_5_) changed, the average value of SDV increased from 2.5825 to 2.755, an increase of 6.3%, indicating that the stearic acid content has little effect on the particle size homogenization of the composite powder. In summary, ball milling time (X_1_), ball milling speed (X_2_), and ultrasonication time (X_4_) are the three main factors affecting the particle size of the composites. In the following section, the effects of ultrasonication time (X_4_), ball milling time (X_1_), and ball milling speed (X_2_) on the properties of the composites and the mechanisms are further analyzed.

### 2.4. Powder Metallurgy (PM)

The composite powders with different parameters set by the orthogonal test were cold-pressed in a mold with a size of φ50 mm × 3 mm by a hydraulic press machine (WAW-600, Jinan New Times Assay Instrument Co., Ltd. Jinan, china) at atmospheric temperature. The pressure was 500 MPa, the compression rate was 2 KN/min, and the holding time was 5 min. And then, the cold-pressed billets were hot-pressed in a vacuum thermocompressor (ZRC85-25T, Jinan Shanda Nonferrous Metal Casting Co., Ltd. Jinan, China) at 600 °C for 1 h with a pressure of 30 MPa supplied by another mould with the diameter of φ50 mm and then cooled in the furnace. Pure aluminium billet was also fabricated by the same processes.

### 2.5. Mechanical Property Tests and Microstructural Experiments

**Figure 4:**
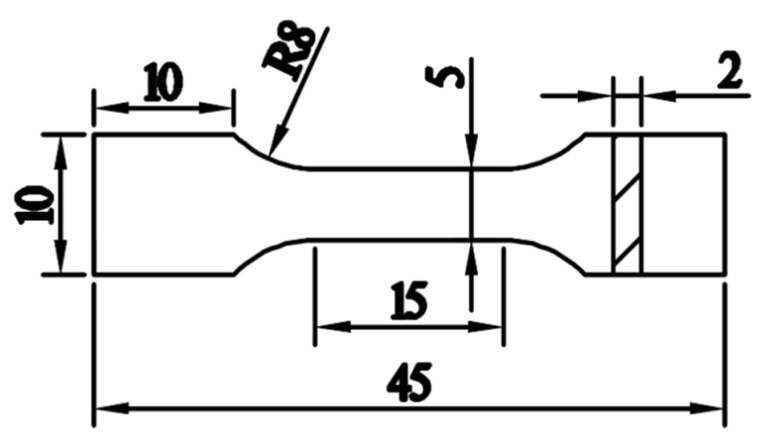
Sizes of tensile samples (unit mm).

Samples were wire electrode cut from the hot-pressed billets of the composite and the pure Al billets for tensile testing, optical microscopy (OM), and SEM scanning. The size of the tensile samples [28,29] is shown in Figure 4. The tensile tests were carried out on a universal testing machine (WDW-5G, Jinan Hengsi Shanda Co., Ltd, Jinan, china) at atmospheric temperature with a tensile rate of 1 mm/min. Three samples for each of the conditions were tested for the repetition.

A field emission SEM (Sigma-300, Carl Zeiss AG, Oberkochen, Germany) was used to observe the morphology of the composite powder after milling and the tensile fracture morphology of the hot-pressed billets and to analyze the element of the composite (EDS). A Raman spectrometer (Renishaw-2000 Renishaw, London, UK) was used to test the components of the graphene, composite powders, cold-pressed, and the hot-pressed composite billets. X-ray diffraction (D/max-2500/PC, Rigaku, Japan) was used to analyze the components of the composite after vacuum hot pressing.

## 3. Results and Discussion

The effects of the three major fabrication parameters on the properties of the 0.5 wt.% GNP/Al composites were studied via comparison of the macroscopic mechanical properties or microscopic characterization.

### 3.1. Effect of Ultrasonication Time (X_4_) on the Dispersion of Graphene

**Figure 5:**
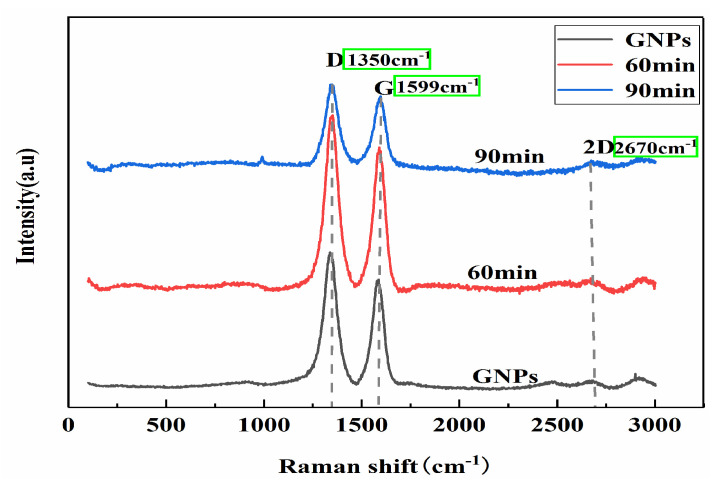
Raman spectra of graphene before and after ultrasonication.

Graphene has many surface atoms and dangling bonds and tends to agglomerate together to reduce the surface activity, which has a negative impact on the mechanical properties of the composites. In addition, due to the large density difference and poor wettability of the Al matrix and graphene, it is more difficult for graphene to uniformly disperse in the Al matrix. The polarity of ethanol is similar to that of graphene, and the van der Waals force between ethanol and graphene is greater than that between the graphene’s own layers. So, under the ultrasonic shaking, graphene in ethanol solution can unfold more easily, dispersing and extending without structural destruction. Figure 5 is the Raman spectra of the GNPs before and after ultrasonication. There are three main characteristic peaks in the Raman spectra of graphene, namely, the D peak around 1350 cm^−1^, the G peak around 1580 cm^−1^, and the 2D peak around 2670 cm^−1^. It is known that the smaller the intensity ratio of the D peak to the G peak (I_D_/I_G_), the more complete the graphene, and the lower the intensity ratio of the 2D band to the G band(I_2D_/I_G_), the larger the number of layers. In this study, as shown in Figure 5, the I_D_/I_G_ value of the original graphene in Figure 5 is 1.21, and the I_2D_/I_G_ value is 0.269; the graphene I_D_/I_G_ value is 1.19, and the I_2D_/I_G_ value is 0.283 after ultrasound for 60 min, while the I_D_/I_G_ value is 1.06 and the I_2D_/I_G_ value is 0.622 after ultrasound for 90 min. The I_D_/I_G_ value of graphene decreases while the I_2D_/I_G_ value increases after ultrasound for 60 and 90 min, indicating that the graphene unfolded and the layer number decreased because of the ultrasonic action, and the longer the ultrasonic time is, the better the dispersion of graphene.

### 3.2. Effects of Ball Milling Time (X_1_) and Ball Milling Speed (X_2_)

#### 3.2.1. Microstructural Morphology of the Composite Powder

**Figure 6:**
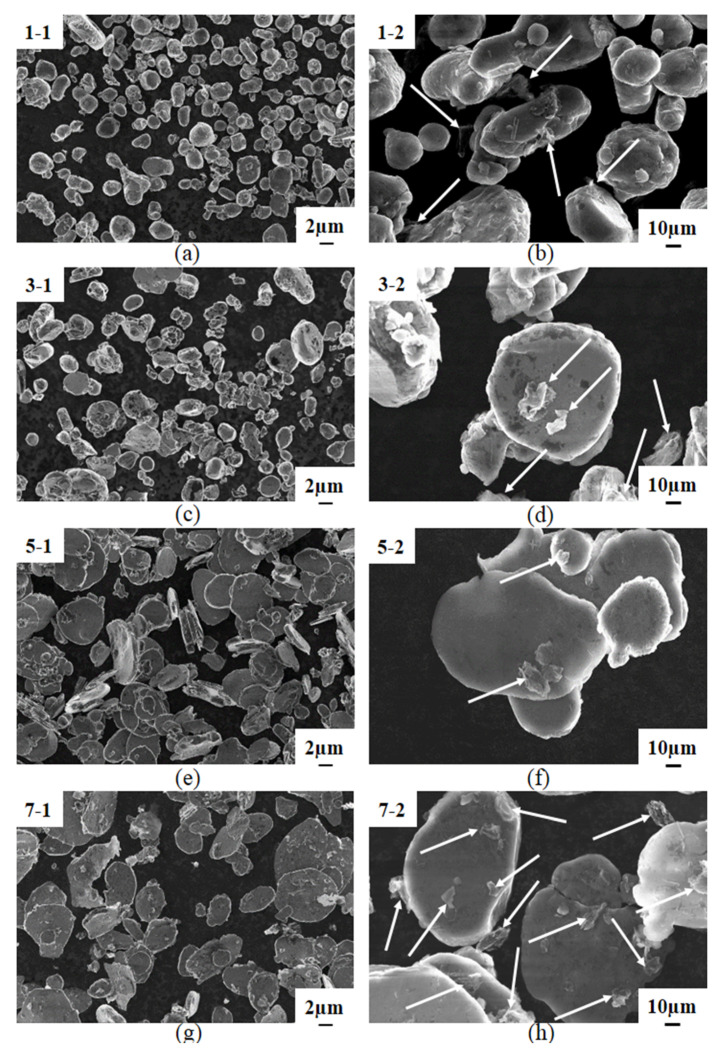

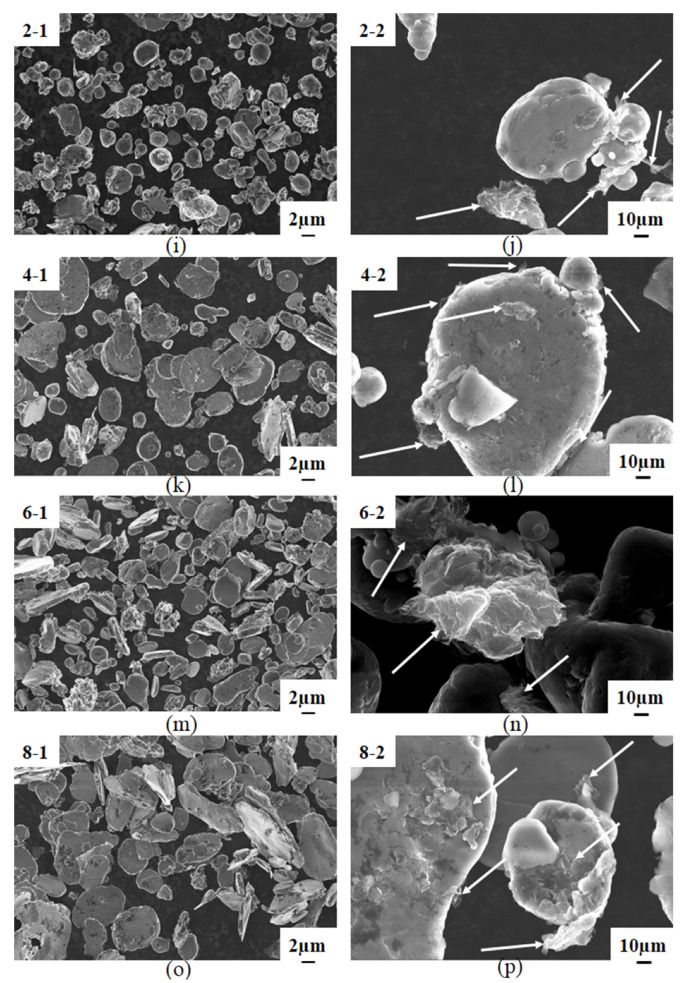
SEM image of the composite powder after ball milling. (**a**–**h**) 200r/min; (**i**–**p**) 300 r/min.

**Figure 7:**
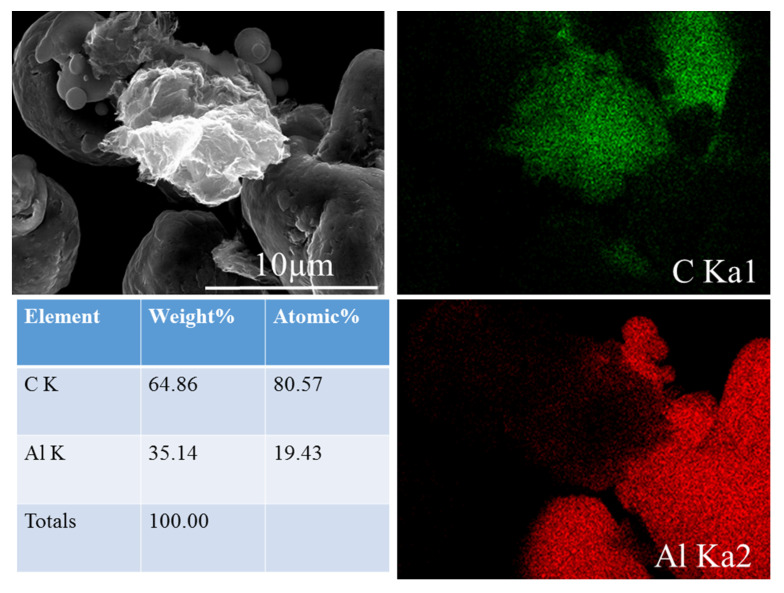
Mapping using EDS.

During high-energy ball milling, the deformation of powder is generally divided into three stages: from spherical to flattened, cold-welded to form larger plate-like particles, and broken to form smaller particles. Meanwhile, graphene was first dispersed on the surface of the flake Al powder and then cold welded into the Al particles. Figure 6 is the SEM image of the composite powder after ball milling. Figure 6a,c,e,g, on the left, are the low-magnification SEM images of the composite correspond to a ball milling speed of 200 r/min and the ball milling time of 2 h, 4 h, 6 h, and 8 h, respectively, and Figure 6b,d,f,h are the high-magnification images. It is the same for Figure 6i–p, but the ball milling speed is 300 r/min. As can be seen from Figure 6a–p, for both of the two milling speeds, as the milling time increases, the Al powder particles gradually change from spherical to flat. And from the comparison of Figure 6a,c,e,g, as well as the comparison of Figure 6i,k,m,o, it can be seen that the uniformity of the powder increases at first and then slopes down to the least when the milling time is 6 h, then becomes larger again when the time reach 8 h, showing the same trend with the SDV values in Table 3. When the milling time is 2 h, there are more free GNPs, as shown by the white arrow in Figure 6b. As the milling time increases, the integrity of the graphene is gradually destroyed to form smaller graphene particles, as shown by the white arrows in Figure 6f,h. However, even when the ball milling time reaches 8 h, graphene fails to form a good bond with the surface of the Al powder, as shown in Figure 6h. At a speed of 300 r/min, when the ball milling time was 6 h, the GNPs were evenly dispersed and partly covered the surface of the Al powder with another part inside the particles, indicating a good infiltration as shown in Figure 6n. This was confirmed by the surface scanning using EDS, as shown in the mapping in Figure 7. When the milling time is 8 h, Al powders become flatter, but at the same time, the GNPs are destroyed, their size is reduced, and the coating is slightly worse than that of the 6 h milling time. This is shown in Figure 6p.

#### 3.2.2. Impact on the Structural Integrity of GNPs

**Figure 8:**
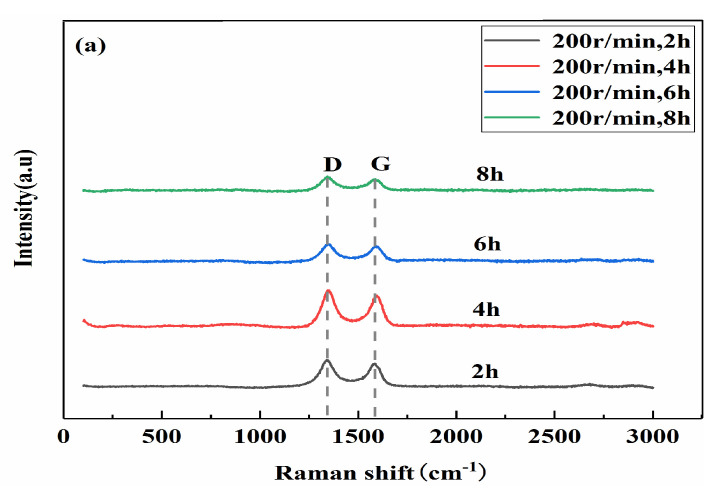

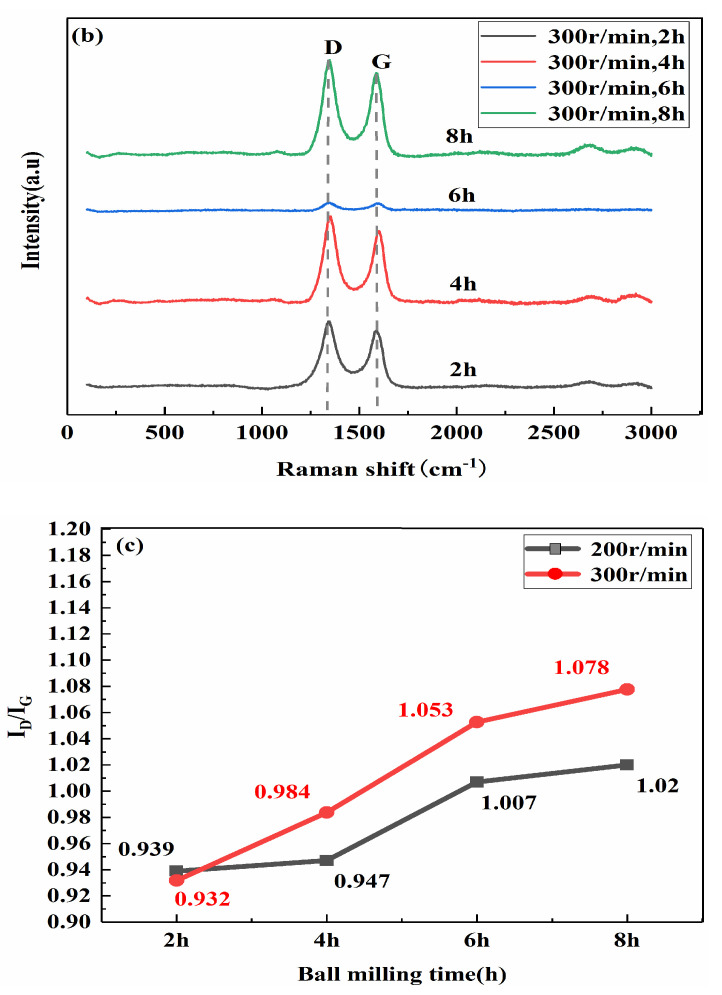
Raman spectra of the composite with different ball milling times. (**a**) Roman shift, 200 r/min; (**b**) Roman shift, 300 r/min; (**c**) I_D_/I_G_.

Figure 8a,b are the Raman diagrams of the composite powder with different ball milling times (X_1_) when the ball milling speeds (X_2_) are 200 r/min and 300 r/min, respectively. Figure 8c shows the change in the I_D_/I_G_ value for 0.5 wt.% GNP composite powders with ball milling times. It can be seen from Figure 8 that, at the same speed, as the ball milling time increases, the value of I_D_/I_G_ gradually increases, indicating that the graphene structure is gradually destroyed. At the same ball milling time, the Raman ratio I_D_/I_G_ with 300 r/min is slightly higher than that at 200 r/min. This is because the high speed will cause a strong impact and a certain degree of damage to the graphene structure. However, the damage degree is limited, and when the ball milling speed is 200 r/min, the coating and bonding between the graphene and the matrix are worse than at 300 r/min, as shown in Figure 6.

#### 3.2.3. Cold-Pressed Composite

**Figure 9:**
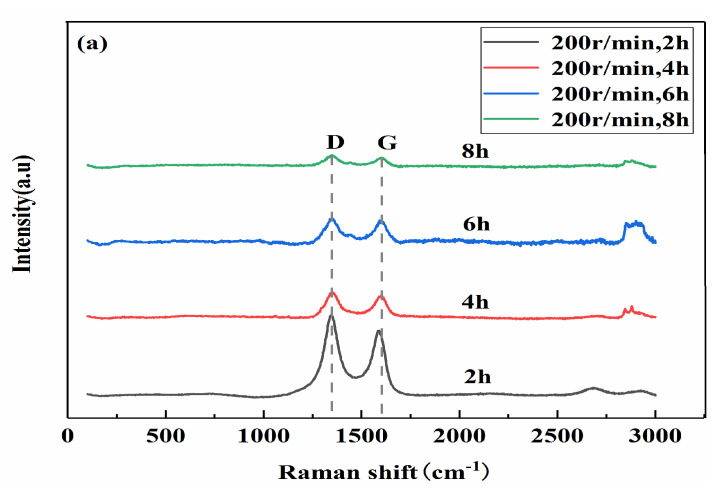

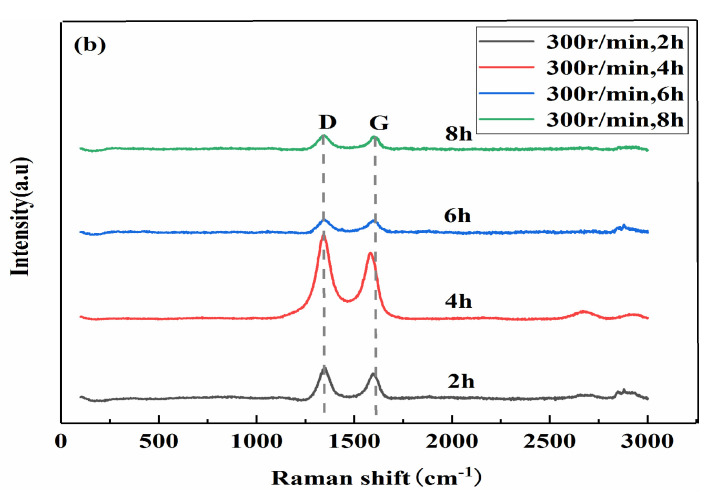
Raman spectrum of the cold-pressed composite. (**a**) 200 r/min; (**b**) 300 r/min.

**Figure 10:**
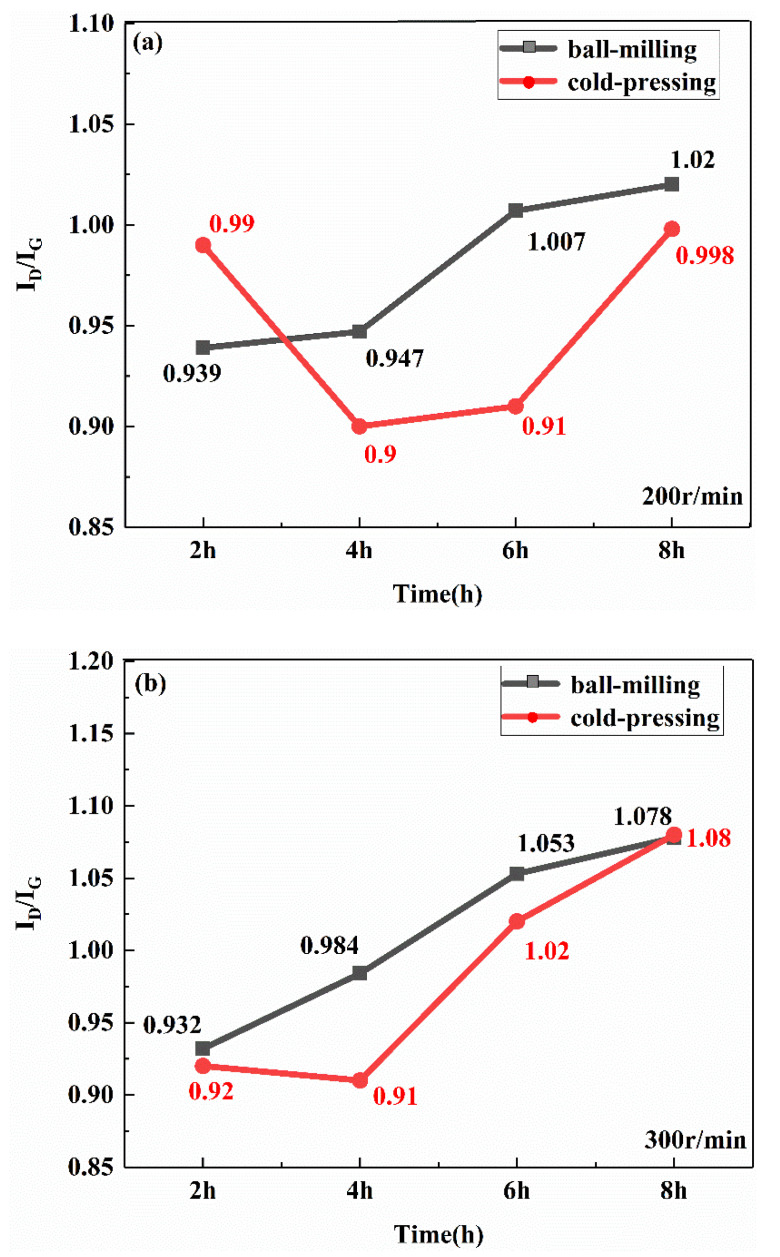
Raman values before and after cold pressing. (**a**) 200 r/min; (**b**) 300 r/min.

Figure 9a,b show the Raman diagrams of the cold-pressed billets with ball milling speeds of 200 r/min and 300 r/min and different milling speeds. Figure 10 shows the comparison of the Raman values before and after cold pressing. Considering the difference in the samples before and after cold pressing, the value difference before and after cold pressing can be ignored, which means that the cold pressing process does not damage the structure of the graphene.

#### 3.2.4. Vacuum Hot-Pressed Composite

**Figure 11:**
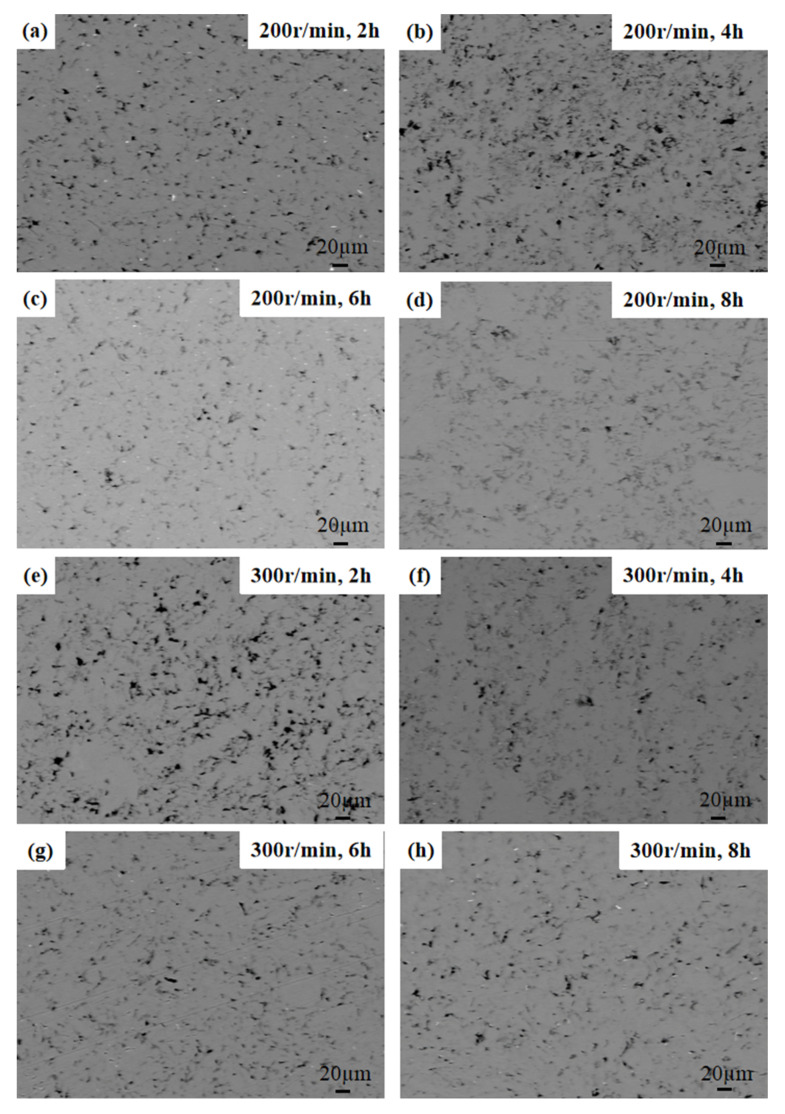
Optical microscope morphology of the vacuum hot-pressed composite. (**a**–**d**) 200 r/min; (**e**–**h**) 300 r/min.

The density of the billet obtained using vacuum hot pressing is approximately 2.69 g/mm^3^. The compactness can reach 99.6%. Figure 11 shows the optical microscope morphology of the vacuum hot-pressed composite. It can be seen clearly that when the ball milling time is 2 h and 4 h, the graphene agglomerates, and the dispersion effect is not as good. When the ball milling time is 6 h, graphene is dispersed uniformly. When the ball milling time is the same, the dispersion of graphene with a ball milling speed of 300 r/min is obviously better than that of the graphene milled at 200 r/min, which shows the same trend as the SDV values in Table 3, verifying that it is reasonable to use SDV to represent the dispersion uniformity of the composites in this paper.

**Figure 12:**
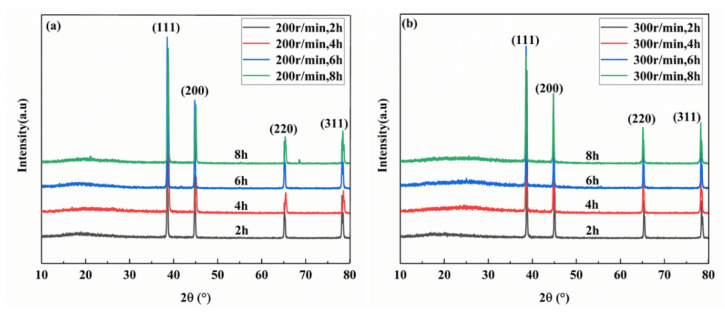
XRD diagram of vacuum hot-pressed composites. (**a**) 200 r/min; (**b**) 300 r/min.

Figure 12 shows the XRD characterization of the hot-pressed composites. There are four peaks, which are all characteristic peaks of Al, and no peaks of graphene and Al_4_C_3_ are detected, which may be due to the low content of graphene and the limited resolution of elements of the X-ray diffraction instrument [30].

#### 3.2.5. Mechanical Properties and Fracture Mechanism

**Figure 13:**
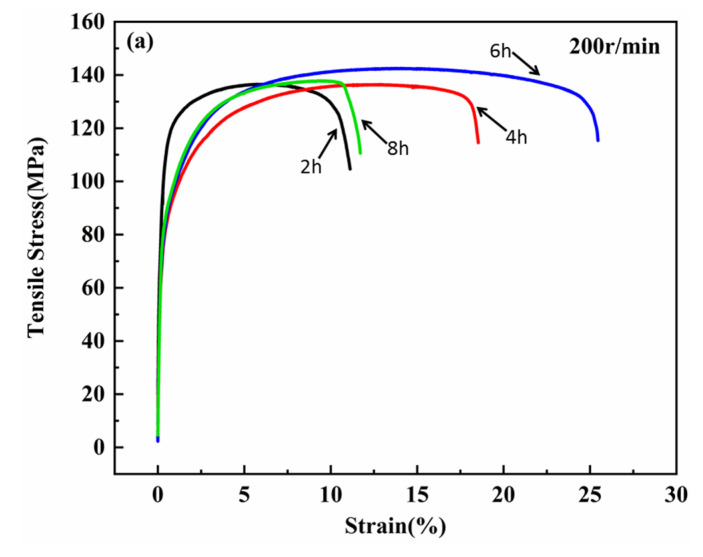

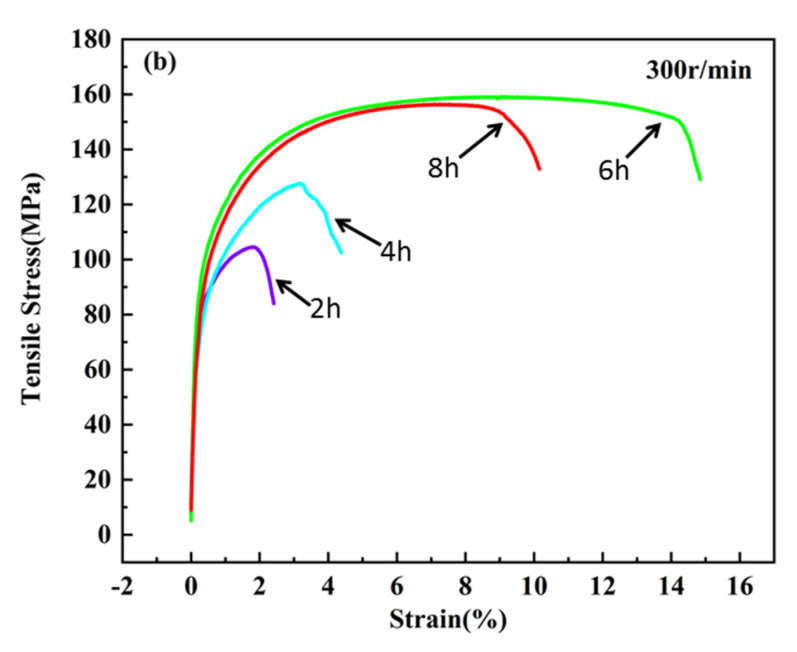
Tensile strength of the hot-pressed composite. (**a**) 200 r/min; (**b**) 300 r/min.

**Figure 14:**
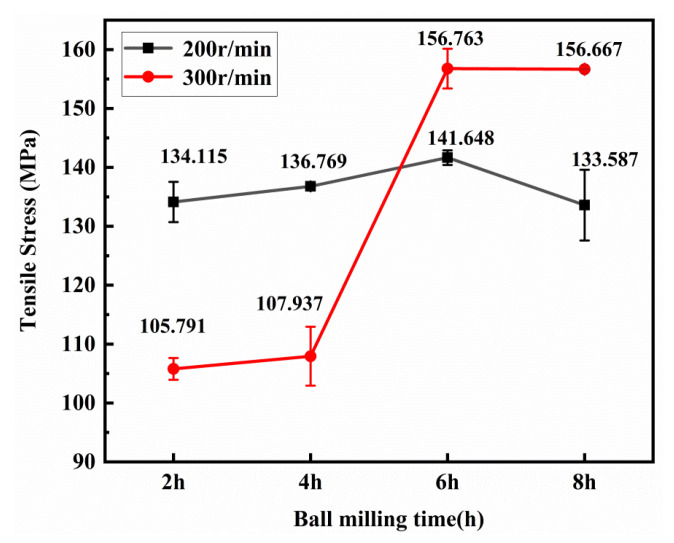
Tensile strength of the composite.

**Figure 15:**
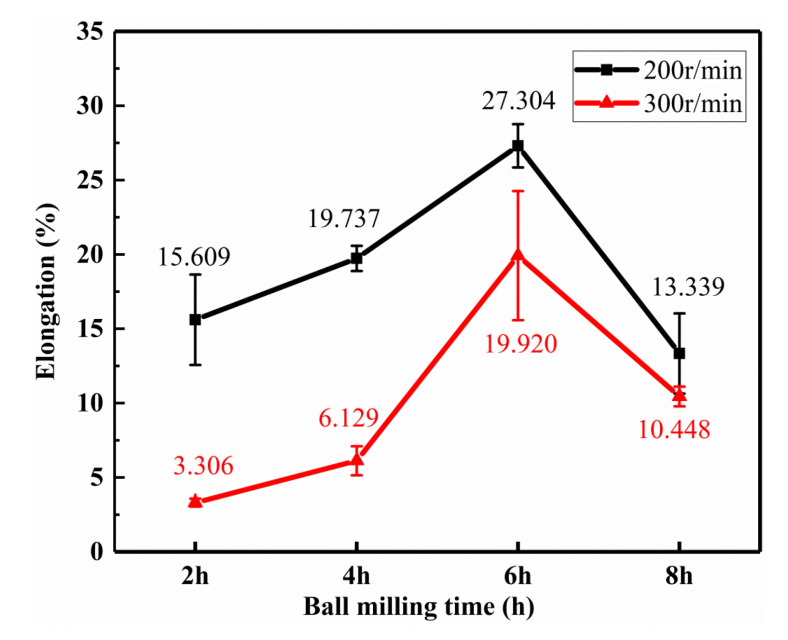
Elongation of the composite.

Figure 13 shows the tensile curve of the 0.5 wt.% GNP/Al composite. Figure 14 and Figure 15 show the mean tensile strength and the mean elongations of it. It can be seen from Figure 13a,b that, for the ball milling speed of 200 r/min and 300 r/min, the tensile strength increases first with the increase of the milling time as a whole. For 200 r/min, when the ball milling time is 6 h, the tensile strength reaches the largest value of 141.7 MPa with the largest elongation at the same time. For 300 r/min, when the ball milling time is 6 h, the elongation of the composite reaches the maximum value of 19.9%, which is 39.82% lower than that of pure aluminum fabricated by the same process in this paper (33.1%), and the tensile strength is 156.8 MPa, which is 56.6% higher than that of pure aluminum (100.5 MPa). When the ball milling time is short, the agglomerate graphene may act as the crack initiation, so the tensile strength and the elongation are poor. When the milling time is appropriate, the graphene can work the best, while if the time is too long, the graphene structure will be destroyed, decreasing the strength and the toughness of the composite [31].

**Figure 16:**
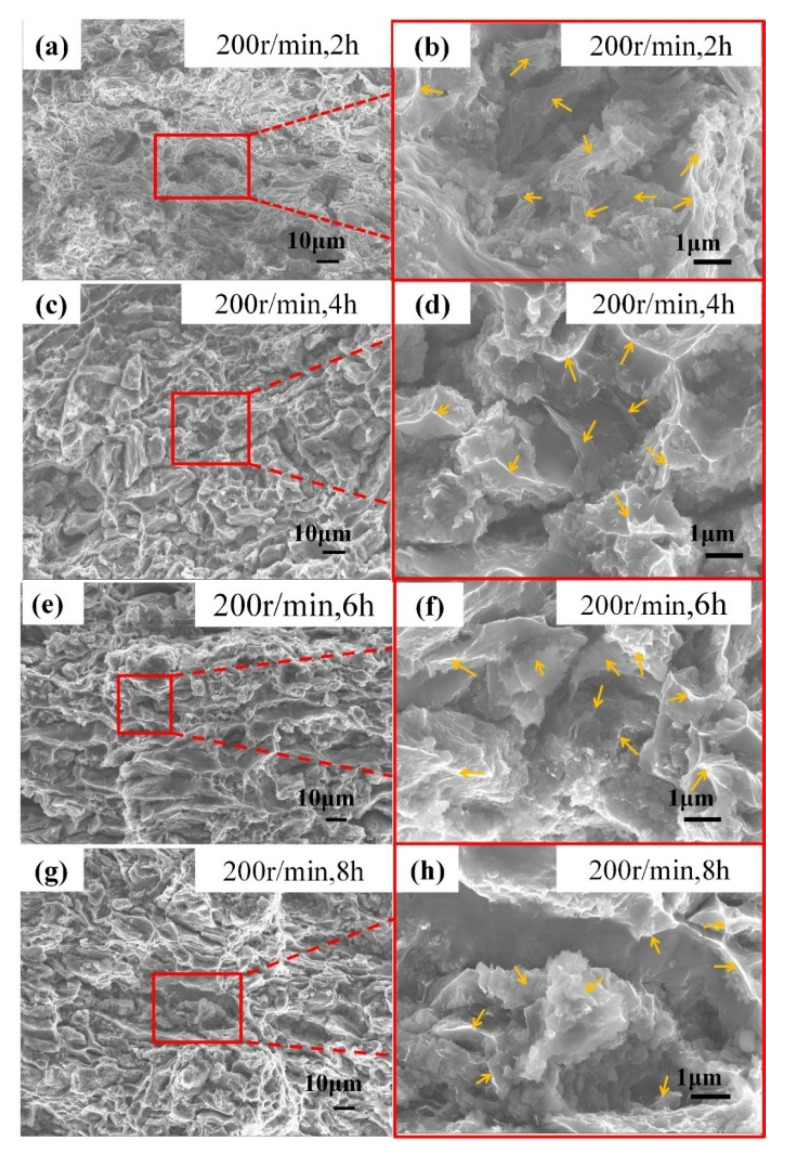

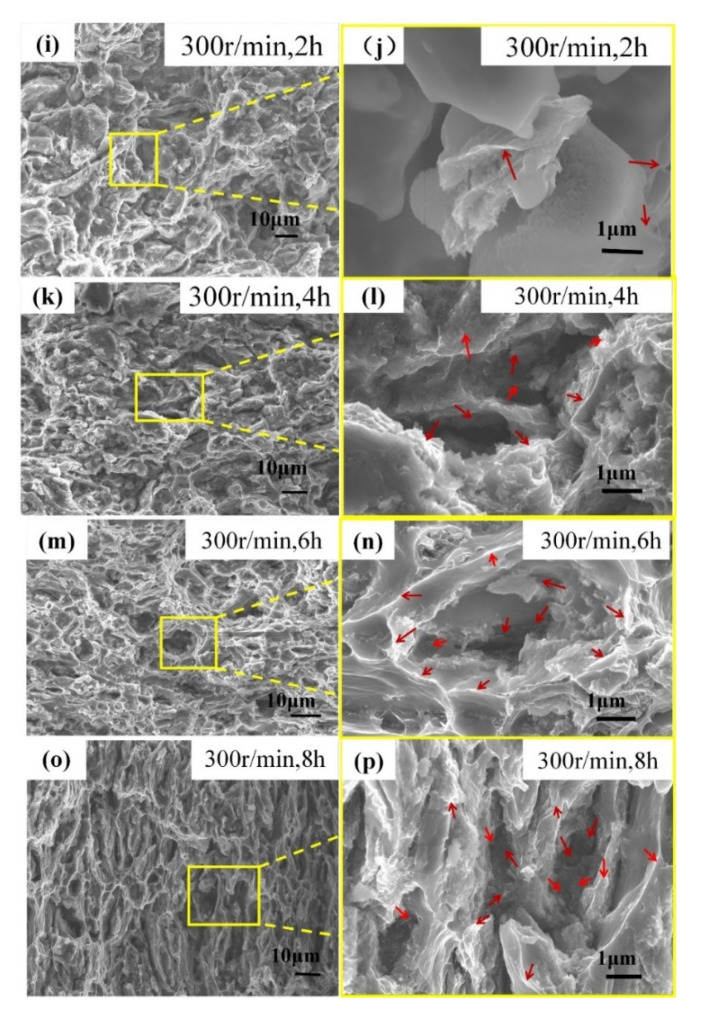
Tensile fracture morphology of the 0.5 wt.% GNP/Al composites.

Figure 16 shows the tensile fracture morphology of the composite. Low-power SEM is on the left side of Figure 15, and the corresponding high-power SEM is on the right. The arrows indicate GNP. The interface bonding between aluminum and graphene is very important to the properties of the composites. The failure mechanisms of the composites mainly include the debonding of the interface, destruction of the reinforcement, and pulling out of the reinforcement. During the process of tensile loading, the agglomerated graphene will act as the crack initiation point, thus affecting the strength of the composites [32]. From Figure 16a,c,e,g, it can be seen that when the ball-milling speed is 200 r/min, with the increase of ball milling time, the fracture surfaces almost have the same morphology: a few large and deep dimples with many small dimples around them. The torn edges are not bright. Figure 16b,d,f,h are the high power magnification near the large dimples. It can be seen clearly that there is discrete and integrated graphene existing inside the relatively larger dimples, which is homologous to the tensile load, and its interface with the matrix is not so good and act as the crack initials. The graphene with good interface of the aluminum matrix bears the most load and forms the bright torn edges. From Figure 16i,k,m,o, it can be seen that when the ball milling speed is 300 r/min, with the increase of the ball milling time, the dimple in the fracture surface gradually becomes larger and deeper, with embedded GNP along the torn edge; at the same time, a large number of small dimples with bright torn edges are distributed around the large dimples, indicating the interface bonding between the matrix and the GNP is perfect. From Figure 16j, when the ball milling time is 2 h, it can be seen that the interface between GNP and alumnium is poor. The fracture tends to be a brittle fracture whose dimples have almost no tearing of the edges. From Figure 16k,m, when the ball milling time is 4 h and 6 h, there is seldom large and discrete graphene, indicating a better embedding between the graphene and the matrix. It can be seen that there are many larger and deeper dimples in the fracture surface, and the torn edges are bright and thin. Therefore, the composite has the best toughness and almost the largest tensile strength. From Figure 16o,p, the ball milling time is 8 h, and the dimple morphology is elongated, which indicates that the maximum principal stress of the material is along the direction of the cross-section. The tensile strength and the toughness begin to decrease. In summary, from the comparison of the particle size and the morphology of the composite powder as well as the mechanical properties and the microstructure of the composite, the ball milling speed of 300 r/min and the ball milling time of 6 h are the best conditions used in this study. This is basically consistent with the results of Xiao et al. [33]. Besides, the graphene ultrasonic time was 90 min, and the stearic acid content was 1.5%.

### 3.3. Fracture Mode Analysis

**Figure 17:**
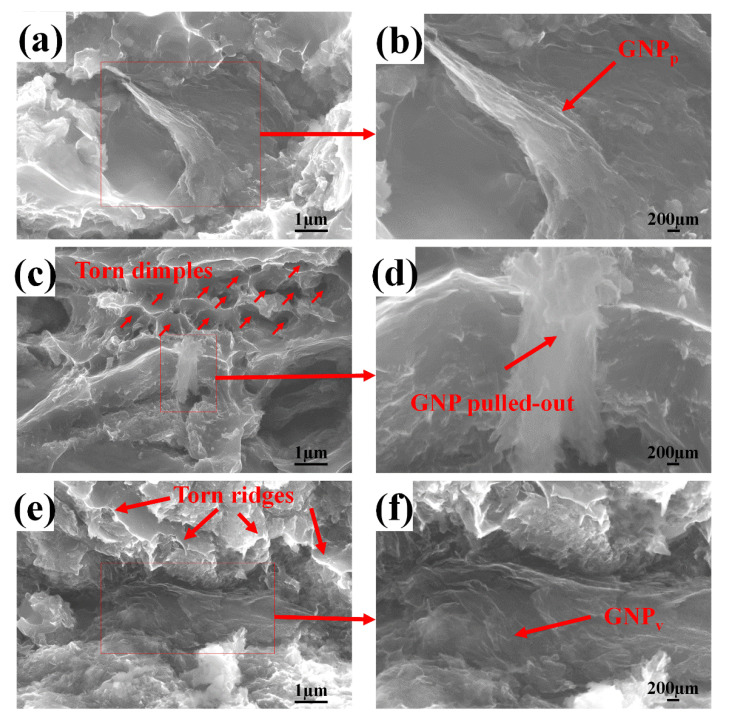
GNP on the tensile fracture surface of the composite. (**a**,**b**) Parallel GNP; (**c**,**d**) pulled-out GNP; (**e**,**f**) vertical GNP.

To analyze the role of the graphene in the reinforcement of the composite, its distributions on the tensile fracture surface are compared in Figure 17. In the composite, the two-dimensional graphene sheet is perpendicular to or parallel to the stretching direction or somewhere intervenient. When the two-dimensional plane of graphene is parallel to the stretching direction (denoted as GNP_p_ in Figure 17a,b), the graphene sheets have the largest contribution to the strength of the composite, whose folds are spread, and then some of them are broken and some are pulled out of the matrix, as shown in Figure 17b,d. The folds spread and broken graphene can effectively bear most of the load, and a large number of torn dimples appear, as shown in Figure 17c, thus improving the tensile strength of the composites effectively [34]. When the two-dimensional plane of the graphene is vertical to the direction of tensile force, (denoted as GNP_v_ in Figure 17e,f), the intermolecular force between graphene sheets is small, the crack initiates on the interlayer of graphene, and the load is mainly borne by the Al matrix, so that a large number of sharp torn edges appear as a cliff on the fracture surface. Thus, the force that the composite can bear is greatly reduced, as shown in Figure 17e, in which the strength of graphene is far from being utilized [34,35]. Therefore, for two-dimensional reinforcement with material such as graphene, the issue of how to reduce its agglomeration and how to achieve uniform dispersion in the matrix is particularly important. For some materials with obvious high-performance in a special dimension, the two-dimensional lamellar direction of graphene should be oriented to be consistent with the stress direction using subsequent processing, such as extrusion, which will be discussed in another paper.

## 4. Conclusions

In this paper, 0.5 wt.% GNP/Al composites were prepared using ultrasonication + ball milling + cold pressing + vacuum hot pressing. The microstructure of 0.5 wt.% GNP/Al created by different fabrication parameters was systematically studied. The main conclusions are as follows:

(1) The density of 0.5 wt.% GNP/Al prepared by cold pressing + vacuum hot pressing is high, and the interface bonding is perfect. No obvious oxidation phenomenon and Al_4_C_3_ were found in the composite. Graphene distributed on the grain boundaries can effectively hinder the growth of the grain during the vacuum hot-pressing sintering and play a role in grain refinements.
(2) Ball milling time, ball milling speed, and ultrasonic time have great influences on the performance of 0.5 wt.% GNP/Al composites.
(3) Adding 0.5% percentage of graphene in weight can obviously increase the strength of the Al matrix. When the ball milling speed was 300 r/min, the ball milling time was 6 h, the B-P ratio was 5:1, the graphene ultrasonic time was 90 min, and the stearic acid content was 1.5%, the graphene nanoplates were uniformly distributed in the Al matrix without being destroyed. The composite had the best comprehensive mechanical properties, with a tensile strength of 156.8 MPa, which is 56.6% higher than that of pure aluminum fabricated by the same process (100.1MPa), while the elongation is 19.9%, which is 39.8% lower than that of pure aluminum (33.1%).

## Figures and Tables

**Table 1:** Orthogonal distribution of the fabrication parameters.

Numbering	Time (h)	Speed (r/min)	B-P Ratio	Ultrasonicatinnon Time (min)	Stearic Acid Content (%)
1	2	200	5:1	60	1.5
2	2	300	10:1	90	3
3	4	200	5:1	90	3
4	4	300	10:1	60	1.5
5	6	200	10:1	60	3
6	6	300	10:1	90	1.5
7	8	200	5:1	90	1.5
8	8	300	5:1	60	3

**Table 2:** Factors in the orthogonal test and their levels.

Level	Factors				
	X_1_ (h)	X_2_ (r/min)	X_3_	X_4_ (min)	X_5_ (%)
1	2	200	5:1	60	1.5
2	4	300	10:1	90	3
3	6	-	-	-	-
4	8	-	-	-	-

**Table 3:** Visual analysis results.

Factors	Sum of SDV				Mean Value of SDV			
	Level 1	Level 2	Level 3	Level 4	Level 1	Level 2	Level 3	Level 4
X_1_	0.78	4.0	0.11	3.55	0.39	2.0	0.055	1.775
X_2_	14.07	8.26	-	-	3.52	2.065	-	-
X_3_	11.61	10.70	-	-	2.90	2.675	-	-
X_4_	8.75	13.6	-	-	2.19	3.40	-	-
X_5_	10.33	11.02	-	-	2.58	2.76	-	-
